# Weed Discrimination at the Seedling Stage in Dryland Fields Under Maize–Soybean Rotation

**DOI:** 10.3390/plants15071114

**Published:** 2026-04-03

**Authors:** Yaohua Yue, Anbang Zhao

**Affiliations:** 1 College of Engineering, Heilongjiang Bayi Agricultural University, Daqing 163319, China; yueyaohua2020@163.com; 2 College of Information and Electrical Engineering, Heilongjiang Bayi Agricultural University, Daqing 163319, China

**Keywords:** maize–soybean rotation, seedling-stage weed detection, YOLOv11n, dynamic convolution, lightweight architecture, the cascaded group attention mechanism

## Abstract

Under maize–soybean rotation systems, weeds and crops at the seedling stage in dryland fields exhibit high similarity in morphological structure, scale distribution, and spatial arrangement. In addition, complex illumination conditions, occlusion, and background interference further complicate accurate weed discrimination. To address these challenges, this study proposes an improved YOLOv11n-based weed detection method for seedling-stage crops under dryland rotation conditions, aiming to enhance detection accuracy and robustness in UAV-acquired field images. Three key improvements were introduced to enhance model performance: (1) the incorporation of Dynamic Convolution (DynamicConv) to adaptively strengthen feature representation for weeds with varying morphologies and scales in low-altitude remote sensing imagery; (2) the design of a SlimNeck lightweight feature fusion architecture to improve multi-scale feature propagation efficiency while reducing computational cost; (3) the cascaded group attention mechanism (CGA) is integrated into the C2PSA module, thereby improving discrimination capability under complex background conditions. These results represent consistent improvements over baseline models, including YOLOv5, YOLOv6, YOLOv8, YOLOv11, and YOLOv12. Specifically, detection performance for broadleaf weeds and Poaceae weeds reached mAP@0.5 values of 87.2% and 73.9%, respectively. Overall, the proposed method demonstrates superior detection accuracy and stability for seedling-stage weed identification under rotation conditions, providing reliable technical support for variable-rate herbicide application and precision field management.

## 1. Introduction

Corn–soybean rotation is one of the most important cropping systems in northern China, with significant advantages in improving soil fertility, reducing pests and diseases, and optimizing cropping structure. Nevertheless, weed infestation at the seedling stage remains one of the key biological stresses restricting crop yield and quality under this rotation system [1]. Weeds not only compete with crops for nutrients, water, and light, but also serve as intermediate hosts for pests and diseases, thereby directly inhibiting seedling growth and subsequent yield. Moreover, weeds reduce the appearance quality of agricultural products during harvesting and cause economic losses. At present, field weed control mainly relies on pre-emergence closed herbicides and post-emergence broadcast spraying. Although weed damage is controlled to a certain extent, this practice leads to problems such as excessive herbicide usage, environmental pollution, and increased production costs [2]. Therefore, developing accurate and efficient weed detection methods for seedling crops under rotation systems, and further realizing on-demand site-specific spraying, is of great significance for promoting sustainable field management and analyzing weed distribution patterns [3,4].

Traditional weed recognition mainly relies on manually designed features (e.g., texture, shape, color) combined with machine learning classifiers such as Support Vector Machine (SVM) [5]. Although such methods are computationally simple, feature extraction highly depends on subjective experience, involves time-consuming design, and has limited generalization ability. Thus, they struggle to adapt to complex field environments, including varying illumination, crop occlusion, and diverse weed morphologies [6].

In recent years, deep learning, especially object detection technology, has been widely used in agricultural vision tasks due to its powerful end-to-end feature learning capability [6,7,8,9]. As a representative one-stage detector, the YOLO series achieves an excellent balance between speed and accuracy, and has been successfully applied to weed detection in various crops, including corn and soybean [10]. For soybean fields, researchers have proposed improved versions based on YOLOv8 and YOLOv7, such as the lightweight YOLOv8-ECFS (with mAP up to 95.0%) [11], YOLO-SW integrating Swin Transformer and RT-DETR for real-time detection [12]. These models significantly improve precision and efficiency for seedling weed recognition in soybean fields. For corn fields, improved YOLOv11 variants such as SLD-YOLO11 and AGRI-YOLO have made progress in fine-grained corn-weed discrimination under complex field conditions [13,14]. Unmanned Aerial Vehicle (UAV) remote sensing further improves data acquisition efficiency. Many studies have combined UAV imagery with modified YOLO models to realize real-time weed monitoring and spatial distribution mapping, such as improved YOLOv11n for small-target detection and density map generation in soybean seedlings [15,16].

Nevertheless, under the corn–soybean rotation system, seedlings of crops and weeds (especially broadleaf and Poaceae weeds) are highly similar in morphology, color, and spatial distribution. Meanwhile, complex field backgrounds, small target sizes, severe mutual occlusion, and imbalanced class distribution bring great challenges to visual recognition. Most existing studies focus on single-crop scenarios, and insufficient attention has been paid to weed detection and generalization across corn–soybean rotation stages. In addition, many models still suffer from bottlenecks, including missed detection of small targets, degraded accuracy for fine-grained weed classification, insufficient robustness under heavy occlusion, and limitations in lightweight real-time deployment. These limitations restrict the practical application of precision weeding technology. In particular, existing unmanned aerial vehicle (UAV)-based weed monitoring approaches largely rely on conducting large-scale flights each year to acquire data and perform real-time analysis. [17]. However, within the relatively stable maize–soybean rotation system, it is feasible to construct task-specific datasets and develop early-stage weed detection models tailored to rotation characteristics, thereby providing a more reliable basis for within-season weed assessment. It should be noted that this study is based on data from a single growing season, which is insufficient to support definitive conclusions regarding weed distribution patterns and their long-term dynamics. Nevertheless, the proposed approach provides a preliminary technical framework for subsequent studies. In the future, with the continuous accumulation of multi-season rotation data and further model optimization, reliance on extensive annual UAV surveys may be reduced. Instead, weed conditions could be inferred by integrating historical rotation information with limited monitoring data, thereby improving the efficiency of variable-rate herbicide application and promoting sustainable agricultural management.

To this end, this study focuses on the accurate identification of seedling weeds under corn–soybean rotation. We establish a UAV-based weed image dataset covering both corn and soybean seedling stages with fine-grained annotations for two weed categories. Furthermore, we propose an improved model based on YOLOv11n, named DSC-YOLOv11n, to achieve efficient and accurate weed classification. The main contributions of this paper are as follows:

To improve weed detection performance in complex field environments, three key improvements are introduced based on YOLOv11n. First, DynamicConv is adopted to adaptively adjust convolution kernel weights, enhancing feature representation for weeds with different shapes and scales, thereby improving the detection of small targets and morphologically diverse weeds [18]. Second, a lightweight feature fusion structure named SlimNeck-2 is designed to optimize multi-scale feature transmission, reduce redundant computation, and maintain multi-scale detection capability, achieving a better trade-off between accuracy and efficiency. Third, the cascaded group attention mechanism(CGA) module is integrated to strengthen the model’s attention to key regions and effective semantic information, improving discrimination ability under complex backgrounds. Through the above improvements, the proposed DSC-YOLOv11n maintains lightweight characteristics while enhancing feature expression and contextual understanding, providing an effective solution for the classification of high-density small-target weeds.

## 2. Materials and Methods

### 2.1. Data Acquisition and Dataset Construction

#### 2.1.1. Data Acquisition

To construct a real-world field dataset suitable for seedling-stage weed recognition research under the corn–soybean rotation system, experimental data were collected from representative corn and soybean test fields in Jianshan Farm, Heilongjiang Province, China. This region has long implemented a corn–soybean rotation cropping system, which improves soil nutrient structure and reduces continuous cropping obstacles through alternate planting of different crops, thereby maintaining the stability of the farmland ecosystem. The test plots are characterized by typical black soil, providing favorable agricultural production conditions.

**Figure 1:**
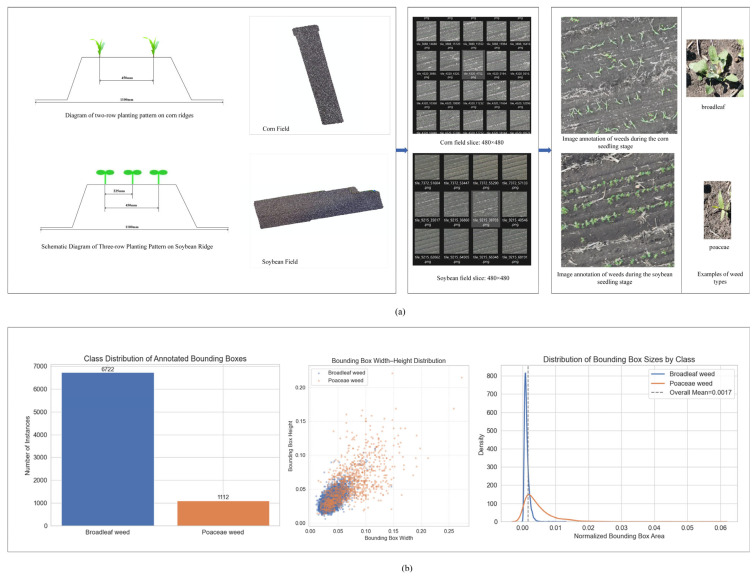
Data set acquisition and construction. (**a**) Corn and soybean field planting patterns and image acquisition; (**b**) Analysis of dataset labels.

Regarding the planting pattern, soybeans are cultivated using the common “three rows on a ridge” method in Northeast China, where three rows of soybeans are planted equidistantly on large ridges approximately 1.1 m wide. Corn is planted using the “two rows on a ridge” pattern, with two rows of corn on large ridges of the same width. The row spacing for both crops is 450 mm. This planting method ensures ventilation and light transmission for crops while optimizing the rationality of the field population structure and adapting to local mechanized operation requirements. To enhance crop growth conditions and yield potential, key agronomic measures such as subsoiling, precision seeding, and side-deep fertilization were implemented during field management to improve soil structure and increase fertilizer use efficiency. The specific planting patterns are illustrated in Figure 1a.

For weed management, no artificial or mechanical weeding was conducted during the experiment to obtain weed image data reflecting real field ecological characteristics, allowing weeds to grow under natural conditions and form a typical field weed community structure. The dominant weed species in the field include Poaceae weeds and broadleaf weeds.

Image acquisition was conducted from 27 May 2025 to 7 June 2025, covering the critical seedling growth stages of corn and soybean. Specifically, soybeans were mainly at the V1–V3 growth stages, while corn was at the V2–V4 stages. To ensure image quality and minimize the impact of illumination variations, daily data collection was scheduled during two time windows: 08:00–11:00 and 13:00–18:00. The field weather conditions during the experiment were favorable, dominated by clear skies and weak or gentle breezes, effectively avoiding image blur caused by drastic light changes or plant sway, thus ensuring the clarity and consistency of the raw data.

Data acquisition was conducted using the DJI Matrice 3M industrial UAV platform, equipped with a high-resolution professional RGB camera and manufactured by SZ DJI Technology Co., Ltd., Shenzhen, China. During acquisition, the camera was maintained perpendicular to the ground to obtain standard orthophotos and minimize perspective distortion. UAV operations followed a preprogrammed fully autonomous flight path, with key flight parameters set as follows: a constant flight speed of 3.6 m/s, a takeoff speed of 15 m/s, and an operational altitude of 12 m above the ground. The forward and side overlaps were set at 80% and 70%, respectively, using a time-interval vertical shooting mode. This high-overlap configuration was intended to acquire dense image sequences, providing a foundation for the subsequent generation of high-precision orthophotos and three-dimensional point cloud data. Each captured image automatically recorded its corresponding GPS coordinates, providing critical data for future studies on the spatial distribution of weeds and enabling precision herbicide application based on geographic information.

A total of 1003 raw field images were collected for both maize and soybean plots. Each image had a resolution of 5280 × 3956 pixels and was stored in JPG format. The dataset comprehensively captures real-field scenarios of maize–soybean rotation during the soybean seedling stage, clearly including seedling maize and soybean plants, various types of weeds (primarily Poaceae and broadleaf species), soil background, and miscellaneous field debris. The complex and diverse scenes provide a high-quality data source for training and testing object detection models in real agricultural environments. The collected raw images were preliminarily screened to remove non-orthophotos resulting from takeoff, landing, or turning, ensuring the final dataset’s integrity and validity.

#### 2.1.2. Dataset Construction

To meet the input requirements of the model and the computational capabilities of the system, and to improve training efficiency, the high-resolution orthophotos were preprocessed. A sliding-window approach was applied to crop fixed-size sub-images from the original images. Considering that UAV-acquired images under natural lighting are susceptible to variations in illumination and slight attitude fluctuations, some cropped sub-images exhibited blur, incomplete targets, or lacked effective weed instances. To ensure dataset quality, all sub-images were manually screened to remove low-quality or invalid images that did not contain clear weed targets, thereby guaranteeing the validity of subsequent annotation and model training. Ultimately, standard images with a resolution of 480 × 480 pixels were cropped from both the soybean and maize plot regions. Specifically, 1171 sub-images were obtained from the soybean plots, and 1099 sub-images from the maize plots, resulting in a total of 2270 base image samples across both field environments.

Since this study aims to detect weeds at the seedling stage and classify them into fine categories, the images were annotated in the format required for object detection tasks. The selected sub-images were manually annotated using the professional image annotation tool LabelImg. Based on morphological characteristics, weeds were classified into two distinct categories: broadleaf weeds and Poaceae weeds. Precise rectangular bounding boxes were drawn around each target and assigned the corresponding class label. Annotation information for each image, including class and bounding box coordinates, was saved in PASCAL VOC format as XML files, serving as ground truth for model training and evaluation. The data collection and annotation workflow is illustrated in Figure 1.

The final dataset consists of 2270 images with a total of 7834 annotated bounding boxes. As illustrated in Figure 1b, 6722 boxes belong to broadleaf weeds, accounting for 85.8%, and 1112 boxes belong to Poaceae weeds, accounting for 14.2%, with a class imbalance ratio of 6.04. Such a distribution reflects the significant dominance of broadleaf weeds in both soybean and corn fields at the seedling stage in terms of quantity and visibility, which is a common phenomenon in many field weed detection datasets. This is mainly due to the more prominent morphological characteristics and higher density of broadleaf weeds at this growth stage.

The normalized area of bounding boxes presents a right-skewed distribution, with a mean value of 0.0017 and a median value of 0.0009. Small objects with an area smaller than 0.01 account for 95.3%, indicating that weed instances are dominated by extremely small targets. The width and height of bounding boxes are mainly distributed in the range of 0–0.10, and the shapes are close to square or slightly elongated without extreme distortion. The above statistical results reveal the characteristics of small-object dominance and class imbalance in the dataset, which provides a basis for subsequent model design and improvement. To evaluate the model’s generalization capability, the dataset was partitioned into a training set (1816 images), a validation set (227 images), and a test set (227 images) using stratified random sampling at a ratio of 8:1:1, ensuring a balanced representation of the two farmland categories.

#### 2.1.3. Experimental Platform Setup and Training Parameters

All experiments were implemented on a self-configured server equipped with a 64-bit Windows 11 operating system, an Intel i5-13490F processor, and an NVIDIA RTX 5060 Ti graphics card with 16 GB of video memory. The network models were constructed based on the PyTorch deep learning framework, with the detailed environment configuration as follows: PyTorch 2.1.0+cu118, CUDA 11.8, and Python 3.9.15.

The hyperparameters for model training were determined comprehensively according to the hardware configuration, dataset characteristics, and empirical debugging. Limited by the 16 GB graphics memory, the batch size was set to 8. The initial learning rate was set to 0.01, which is suitable for fine-tuning tasks based on pre-trained weights to ensure rapid convergence and avoid training oscillation caused by excessive weight updates. The weight decay was set to 0.001 to mitigate model overfitting and improve generalization ability. All experiments were trained with a single-scale input size of 480 × 480 pixels, consistent with the preprocessed image size of the dataset to ensure data matching.

Considering the lightweight characteristics of the model and the training pattern of the small-target weed dataset, the number of training epochs for both YOLOv11 and the improved YOLOv11 was set to 300 to guarantee sufficient convergence. The YOLOv11 pre-trained weights were adopted, which accelerate training convergence and enhance detection accuracy by leveraging the universal features learned from large-scale datasets.

#### 2.1.4. Evaluation Metrics

To systematically and objectively evaluate and compare the performance of different models on the task of **weed species identification at the seedling stage under maize–soybean rotation**, this study selected key evaluation metrics from two core dimensions: model accuracy and model efficiency. The final performance of all models was calculated and compared on an independent test set to objectively reflect their generalization ability in real, unseen field scenarios.

Model Accuracy Metrics: Accuracy metrics quantify the model’s performance in the weed species identification task and include the following indicators: Precision(Box) and Recall(Box): Precision measures the proportion of predicted bounding boxes that are correctly identified as a specific weed category, reflecting the correctness of the model’s predictions. Recall measures the proportion of actual targets of a specific category that are successfully detected by the model, reflecting the model’s ability to discover all relevant targets. Mean Average Precision (mAP@0.5(Box)): Using an intersection-over-union (IoU) threshold of 0.5, the average precision (AP) is computed for the two categories—broadleaf weeds and Poaceae weeds—and their mean is taken as mAP@0.5. This metric integrates both precision and recall, providing a comprehensive measure of the model’s overall performance in weed detection and classification. It serves as the primary accuracy metric for model comparison in this study. Mathematically, these metrics are defined as follows:

(1) Mean Average Precision (mAP)

(1) $$\mathrm{mAP}@0.5=\frac{1}{N}\sum_{i=1}^{N}AP_{i}$$

where N represents the number of categories in the detection task, and $AP_{i}$ denotes the average precision for the i-th category.

(2) Precision

(2) $$\mathrm{Precision}=\frac{TP}{TP+FP}$$

where TP (true positives) is the number of correctly predicted positive samples, and FP (false positives) is the number of negative samples incorrectly predicted as positive.

(3) Recall

(3) $$\mathrm{Recall}=\frac{TP}{TP+FN}$$

where TP represents the number of true positives correctly identified by the model, and FN (false negatives) is the number of positive samples incorrectly predicted as negative.

Model Efficiency Metrics: Efficiency metrics evaluate the computational complexity and deployment feasibility of the model, focusing on model’s lightweight characteristics and inference speed. Number of Parameters (Params): The total number of trainable parameters in the model. A smaller parameter count generally indicates a more compact model, which is advantageous for deployment on edge devices with limited storage. Floating Point Operations (GFLOPs): The number of billions of floating-point operations required for a single forward pass of the model on an input image. This metric reflects the computational complexity of the model. Lower GFLOPs typically correspond to faster theoretical inference speeds and lower hardware requirements, which is beneficial for real-time field processing.

### 2.2. Weed Detection Model at the Seedling Stage Under Maize–Soybean Rotation

#### 2.2.1. YOLOv11 Detection Model

To develop an accurate detection model for seedling-stage weed identification under maize–soybean rotation, a baseline architecture that balances high precision and efficiency is required. YOLOv11, the latest object detection algorithm released by Ultralytics in 2024, significantly enhances performance through its optimized network design. Its architecture is composed of three core components: a backbone for feature extraction, a neck for feature fusion, and the detection head.

The backbone employs an improved CSPDarknet structure, generating multi-scale feature maps through five downsampling stages and innovatively integrating the C2PSA module. This module incorporates a multi-level Pyramid Slice Attention mechanism, which effectively enhances the model’s ability to capture subtle discriminative features between crops and seedling-stage weeds. The neck uses an enhanced PAN-FPN structure to fuse deep semantic features with shallow positional information through both top-down and bottom-up paths. This design effectively improves the model’s detection capability for small weeds that are densely distributed and exhibit large-scale variations in field environments. The detection head adopts a decoupled design, separating the classification and bounding box regression tasks and optimizing the loss functions for each. The classification branch employs binary cross-entropy loss, while the bounding box regression combines Distribution Focal Loss and CIoU loss, collaboratively improving both classification accuracy and localization precision. Additionally, the use of depthwise separable convolutions in the classification branch significantly reduces the number of parameters and computational complexity. These design choices enable YOLOv11 to maintain high detection precision while supporting real-time inference, meeting the deployment requirements of agricultural mobile platforms or edge devices and providing a robust foundation for further model enhancements in complex farmland scenarios.

#### 2.2.2. Improved YOLOv11 Weed Detection Model

**Figure 2:**
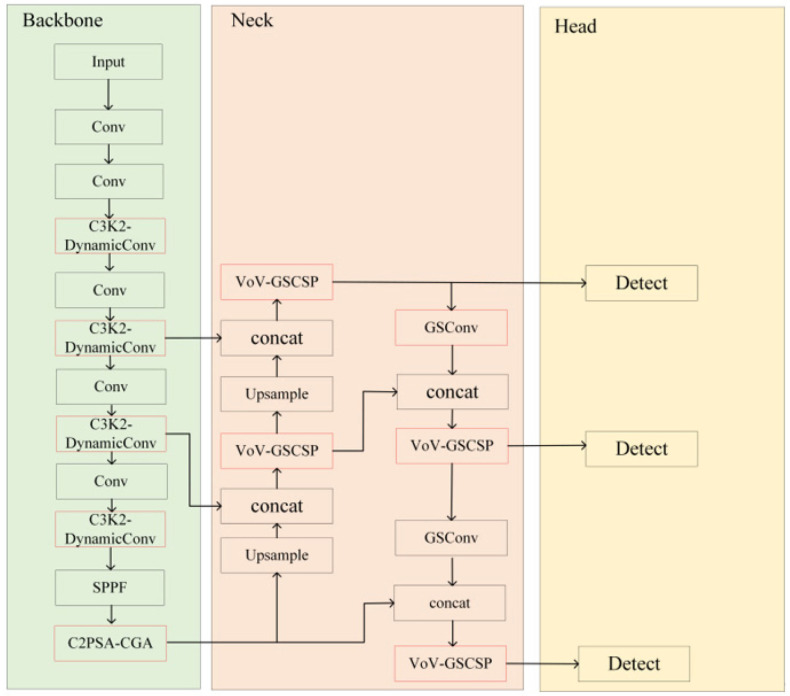
Improved DSC-YOLOv11n model architecture.

To address the challenges of detecting weeds during the seedling stage in maize–soybean rotation dryland—namely highly variable target morphology, significant scale differences, and complex field backgrounds—this study adopts the lightweight YOLOv11n network as a baseline. By systematically integrating three core improvements, namely Dynamic Convolution (DynamicConv), a lightweight neck structure (SlimNeck-2), and Cascaded Group Attention (CGA), we developed DSC-YOLOv11n, a weed detection model that balances high accuracy with computational efficiency. The overall architecture of the model is shown in Figure 2.

The core design of the model lies in integrating these three modules into the YOLOv11n framework, forming a dedicated architecture tailored for weed detection during the maize–soybean seedling stage. Each module contributes distinct but complementary functions: DynamicConv aggregates multiple parallel convolutional kernels through an attention mechanism, enhancing the network’s ability to adaptively extract features from weeds of varying scales and morphologies; SlimNeck-2 optimizes the feature fusion pathway while maintaining a lightweight design, effectively preserving and transmitting fine-grained information of small targets and alleviating feature degradation in deep layers; CGA aggregates the outputs of grouped attention heads in a cascaded manner, capturing rich contextual information while improving computational efficiency, thereby significantly enhancing the model’s capacity to distinguish between grass-like and broadleaf weeds at a fine-grained level.

Through the synergistic integration of these modules, the proposed DSC-YOLOv11n model achieves a substantial improvement in detection accuracy while maintaining excellent computational efficiency, providing a robust technical foundation for precision agriculture operations. In the following sections, the design principles of each improvement module, their integration strategy, and their specific functional roles within the overall network will be described in detail.

#### 2.2.3. C3k2_DynamicConv Module

**Figure 3:**
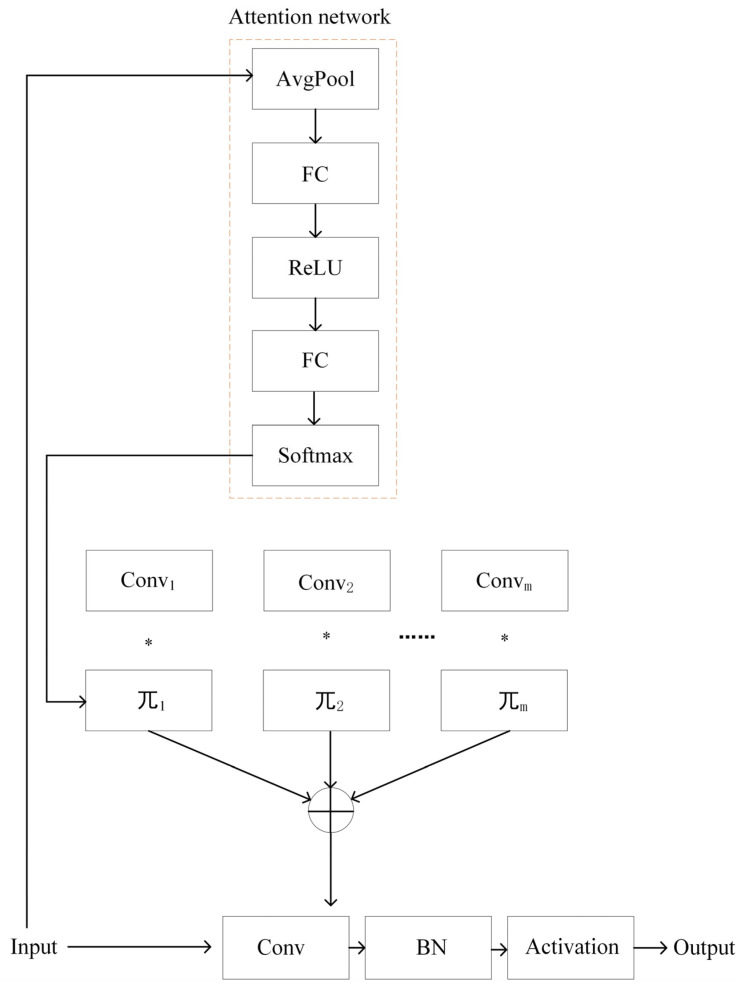
Structure of the DynamicConv Module, **Note:** ${\mathrm{Conv}}_{m}$ denotes the m-th group of convolution kernels; $\pi_{m}$ denotes the weight coefficient of the m-th group of convolution kernels; AvgPool represents the average pooling operation; FC denotes the fully connected layer; ReLU represents the activation function; Softmax denotes the normalization operation; BN represents batch normalization; Activation denotes the activation function; → indicates the data flow; ⋯⋯ represents the parameter flow; ⊕ denotes the summation operation.

In seedling-stage dryland scenarios under maize–soybean rotation, traditional fixed convolution kernels struggle to simultaneously capture diverse visual patterns due to factors such as natural illumination variations, significant scale differences between crops and weeds, and complex field backgrounds. This often results in insufficient feature representation for weed species, leading to recognition difficulties or misclassifications. To address these limitations, a DynamicConv mechanism is introduced into the C3k2 module of the YOLOv11n backbone [19], forming the C3k2_DynamicConv module, which enhances the network’s adaptive feature modeling capability in complex lighting and rotation cropping environments, as shown in Figure 3.

The core idea of DynamicConv is to replace a single static convolution kernel with M base kernels. $\left\{{\mathrm{conv}}_{1},{\mathrm{conv}}_{2},\ldots ,{\mathrm{conv}}_{M}\right\}$, and dynamically fuse them according to input-dependent attention weights, thereby adaptively generating optimal convolution parameters for different input features. The dynamic convolution weights and biases are defined as follows:

(4) $$W_{\mathrm{dyn}}(x)=\sum_{m=1}^{M}\pi_{m}(x)W^{\left(m\right)}$$

(5) $$b_{\mathrm{dyn}}(x)=\sum_{m=1}^{M}\pi_{m}(x)b^{\left(m\right)}$$

(6) $$y=W_{\mathrm{dyn}}(x)\times x+b_{\mathrm{dyn}}(x)$$

where $W_{\mathrm{dyn}}(x)$ and $b_{\mathrm{dyn}}(x)$ denote the dynamically generated convolution weight matrix and bias vector based on input feature x; M is the number of predefined base kernels; $\pi_{m}(x)$ is the attention weight for the m-th kernel; $W^{\left(m\right)}$ and $b^{\left(m\right)}$ are the weight and bias of the m-th base kernel; and * represents the convolution operation.

The attention weights $\pi_{m}(x)$ are adaptively generated by a lightweight attention network, as illustrated by the dashed box in Figure 3. First, the input feature map undergoes Global Average Pooling (GAP) to obtain a channel descriptor vector that reflects global spatial information:

(7) $$z_{c}=\frac{1}{HW}\overset{H}{\underset{i=1}{\sum }}\sum_{j=1}^{W}x_{c,i,j},z\in R^{C_{\mathrm{in}}}$$

where $x_{c,i,j}$ represents the feature value at channel c and spatial location $\left(i,j\right)$; H and W denote the height and width of the feature map; and $C_{\mathrm{in}}$ is the number of input channels.

The vector z is then passed through a two-layer fully connected attention mapping network, with a ReLU activation in the hidden layer to enhance non-linear representation:

(8) $$s=W_{2}\;\mathrm{ReLU}(W_{1}z+b_{1})+b_{2},s\in R^{M}$$

Finally, the output vector s is normalized using a Softmax function to obtain the attention weights. $\pi_{m}\left(x\right)$ for each base convolution kernel:

(9) $$\pi_{m}(x)=\frac{exp(s_{m})}{\sum_{m^{’}=1}^{M}exp(s_{m^{’}})},\pi (x)\in R^{M}$$

where $W_{1}$ and $W_{2}$ are the weight matrices of the two fully connected layers, $b_{1}$ and $b_{2}$ are the corresponding biases, s is the output score vector of dimension M (equal to the number of predefined base kernels), $s_{m}$ denotes the score of the *m*-th branch, and $s_{m^{’}}$ denotes the score of the $m^{’}$ the branch. The Softmax normalization ensures that the attention weights $\pi_{m}(x)$ sum to 1, enabling adaptive weighted fusion of the base convolution kernels according to the input features.

After introducing the DynamicConv mechanism into the C3k2 module, the network can adaptively adjust the combination of convolution kernels according to the differences in scale, texture, and illumination conditions among maize seedlings, soybean seedlings, and various weed species at the seedling stage. In this way, collaborative modeling of diverse local features and global semantic information can be achieved.

This improvement effectively enhances the robustness of the backbone network in feature extraction under complex field environments, while introducing almost no significant increase in the number of parameters. As a result, it provides more discriminative feature representations for the subsequent weed detection head.

#### 2.2.4. SlimNeck Feature Fusion Structure

In the design of lightweight object detection networks, Depthwise Separable Convolution (DSC) has been widely adopted due to its significant reduction in parameters and computational cost. However, excessive use of DSC often weakens inter-channel information interaction, leading to limited feature representation capability and making it difficult to achieve sufficient detection accuracy in complex scenarios. To address this issue, Li Hulin et al. [20] proposed a high cost-performance Neck design paradigm named SlimNeck, which aims to achieve a better speed–accuracy trade-off while maintaining detection performance [21,22].

**Figure 4:**
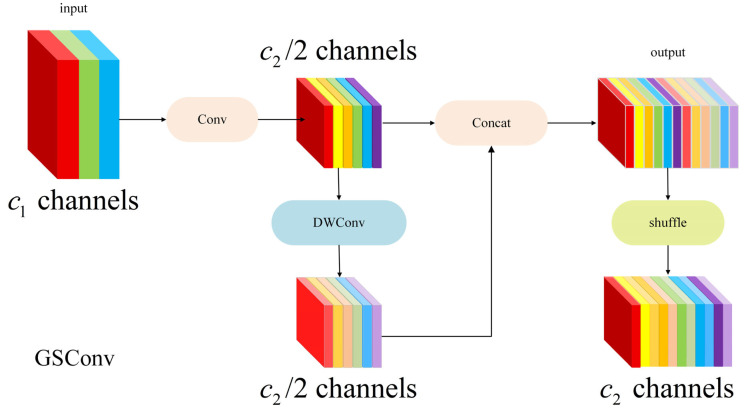
Structure of the GSConv module.

The core of SlimNeck lies in introducing a novel lightweight convolution structure called GSConv (Ghost–Shuffle Convolution), which reduces model complexity while making its output features approximate the representational capability of standard convolution as closely as possible. As shown in Figure 4.

Let the input feature map be:

$$X\in R^{C_{in}\times H\times W}$$

The feature generation process of GSConv can be formulated as follows:

(10) $$Y_{1}=\mathrm{Conv}(X)$$

(11) $$Y_{2}=\mathrm{DWConv}(X)$$

(12) $$Y=\mathrm{Shuffle}(\mathrm{Concat}(Y_{1},Y_{2}))$$

where Conv(⋅) denotes the standard convolution operation, used to obtain high-quality primary feature maps; DWConv(⋅) represents depthwise separable convolution, which supplements feature information at lower computational cost; Concat(⋅) indicates channel-wise concatenation; Shuffle(⋅) denotes the channel shuffle operation, which disrupts channel ordering to enable thorough inter-channel information fusion between features from different convolution paths, thereby enhancing cross-channel interaction capability.

Through this hybrid design, GSConv effectively compensates for the reduced channel interaction caused by pure depthwise separable convolution, while maintaining lightweight characteristics—providing an efficient feature fusion solution for the SlimNeck structure.

Compared with standard convolution, GSConv significantly reduces computational cost while maintaining strong feature representation capability. Its computational complexity can be approximately expressed as:

$${\mathrm{FLOPs}}_{\mathrm{GSConv}}\ll {\mathrm{FLOPs}}_{\mathrm{Conv}}$$

In the Neck structure design, SlimNeck further incorporates the One-Shot Aggregation (OSA) concept to construct a cross-stage partial module named VoVGSCSP, aiming to enhance multi-level feature fusion while reducing redundant computation.

The feature aggregation process of VoVGSCSP can be formulated as:

(13) $$F_{\mathrm{out}}=A(F_{1},F_{2},\ldots ,F_{n})$$

where $F_{i}$ denotes the intermediate feature map of the i-th stage, and $A\left(\cdot \right)$ represents the feature aggregation function.

This design reduces repetitive feature computation while improving feature reuse efficiency, thereby significantly lowering inference time without sacrificing detection accuracy.

**Figure 5:**
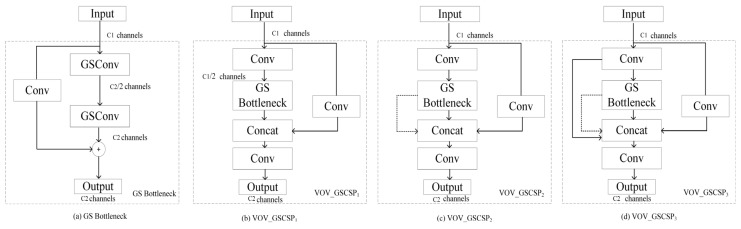
Structure diagram of VOVGSCSP.

As shown in Figure 5, the VoVGSCSP module has three typical structural variants: V1, V2, and V3.

V1 Structure: The simplest design, featuring a shorter inference path and lower latency. It demonstrates higher computational cost-effectiveness in practical deployment scenarios.V2 and V3 Structures: These variants enhance feature reuse capability, further improving feature representation strength. However, they introduce relatively higher computational overhead. Considering the trade-offs among detection accuracy, inference speed, and deployment requirements, this study prioritizes the V2 structure in SlimNeck-2.

In addition, SlimNeck-2 introduces an enhanced module based on GSConv, named GS Bottleneck. By combining the advantages of standard convolution and depthwise separable convolution within a bottleneck structure, it further strengthens feature transformation capability. Its fundamental form can be expressed as:

(14) $$F_{b}=X+\mathrm{GSConv}(X)$$

This residual structure ensures stable gradient propagation while effectively improving the network’s ability to model complex textures and fine-grained edge features.

Considering the practical requirements of weed detection at the seedling stage in maize–soybean rotation dryland conditions, SlimNeck-2 significantly improves multi-scale feature fusion efficiency in the Neck stage of YOLOv11n. This enables the network to more accurately characterize the subtle differences in scale, morphology, and texture between weeds and crop seedlings during early growth stages. Meanwhile, its lightweight design effectively satisfies the low-latency and high-efficiency requirements of UAV-based platforms and real-time field detection applications. As a result, it provides more compact and highly discriminative feature representations for the subsequent detection head [23].

#### 2.2.5. CGA Module

In the weed detection scenario at the seedling stage under maize–soybean rotation dryland conditions, frequent mutual occlusion among crop seedlings, as well as dynamic occlusion between weeds and environmental elements (such as soil, residues, or irrigation facilities), results in severe visual occlusion in the collected image data. This multi-source and dynamic occlusion leads to the loss of critical feature information of weeds and crops, thereby increasing recognition errors of the YOLOv11n model and reducing detection accuracy and robustness. To address this issue, a Cascaded Group Attention (CGA) mechanism is introduced and embedded into the C2PSA module [24]. The CGA module enhances differentiated feature representation across multiple levels through grouped attention and cascaded information transmission. Furthermore, CGA strengthens the correlation between channel and spatial features via cross-head cascading, effectively mitigating performance degradation caused by occlusion. It is particularly suitable for weed detection in complex dryland seedling environments where weeds exhibit diverse morphologies and often overlap with crop leaves.

The core idea of CGA is to divide the input features into multiple attention heads. Each head maps the input features into different subspaces using projection matrices. $W_{Q}^{ij}$, $W_{K}^{ij}$, and $W_{V}^{ij}$, and performs self-attention within each group to generate locally enhanced features. The computation process is formulated as follows:

(15) $${\tilde{X}}_{ij}=\mathrm{Attn}(X_{ij}W_{Q}^{ij},\;\;X_{ij}W_{K}^{ij},\;\;X_{ij}W_{V}^{ij})$$

(16) $${\tilde{X}}_{i+1}=\mathrm{Concat}[{\tilde{X}}_{ij}{]}_{j=1}^{h}W_{P}^{i}$$

(17) $$X_{ij}^{’}=X_{ij}+{\tilde{X}}_{i(j-1)},1<j<h$$

where j denotes the group index after feature partitioning; h represents the total number of attention heads; $\mathrm{Attn}\left(\cdot \right)$ denotes the self-attention operation; $W_{P}^{i}$ is the projection matrix used to restore the concatenated features to the original dimensionality.

**Figure 6:**
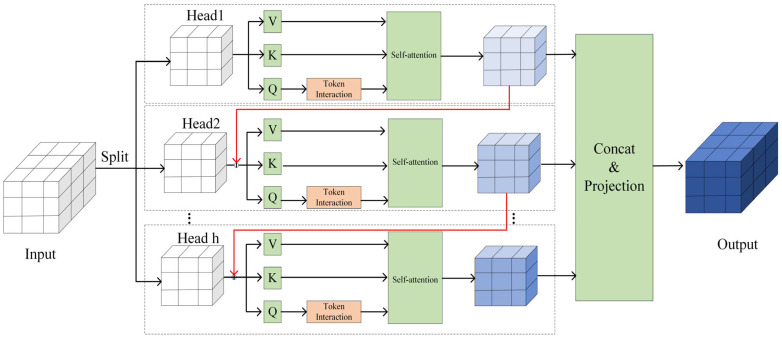
Illustrates the overall architecture of the CGA module. Note: Head1, Head2, and Head h denote different attention heads; Q, K, and V represent the query, key, and value vectors, respectively; Token Interaction denotes secondary feature interaction; Self-attention refers to the self-attention module; Concat & Projection indicates feature concatenation and projection.

Through this cascaded mechanism, inter-group information is progressively transmitted, enhancing global feature extraction capability—especially for recovering lost edge and texture information of weeds in occluded regions. Additionally, after projecting the query vector Q, an extra Token Interaction layer is introduced. By applying depthwise convolution, this layer enables Q to incorporate global contextual information while capturing fine-grained local details. This significantly improves the model’s ability to understand both local relationships (e.g., fine weed leaf textures) and global relationships (e.g., overall crop layout). This is particularly critical in dryland seedling weed detection, where weeds often grow in small clusters and are easily occluded by crops. The network structure of the CGA module is shown in Figure 6.

To enable the model to capture more refined weed characteristics (such as morphology, distribution, and growth state) in complex maize–soybean rotation dryland environments and improve its adaptability to occlusion, the original attention mechanism in the PSABlock of the C2PSA module is replaced with CGA. This modification not only enhances feature robustness but also reduces occlusion-induced noise interference through the grouped cascading mechanism, thereby improving the mean Average Precision (mAP) of weed detection.

**Figure 7:**
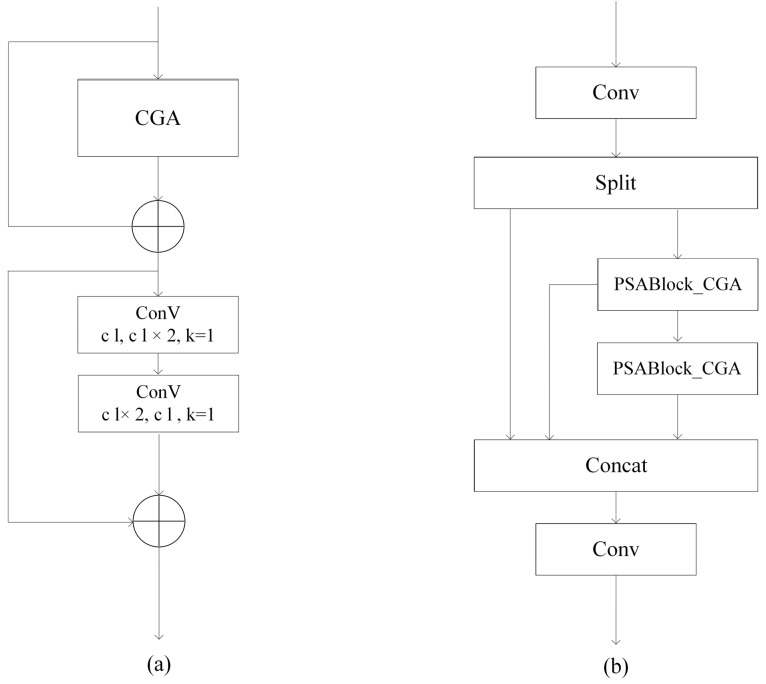
Illustration of the improved C2PSA module. (**a**) PSABlock_CGA; (**b**) C2PSA_CGA.

The PSABlock_CGA (see Figure 7a) module consists of the CGA mechanism and two cascaded 1×1 convolution blocks. Workflow: The input feature first passes through the CGA module to enhance key features (e.g., prominent weed edges). The output feature map is then processed by the first 1×1 convolution for channel expansion, increasing channels from $c_{1}$ to $2c_{1}$. A second 1×1 convolution compresses the channels back to $c_{1}$, ensuring dimensional consistency. To optimize gradient flow and improve feature reuse, two residual connections are introduced: One connects the CGA output with the input of the first 1×1 convolution via element-wise addition. The other fuses the output of the second 1×1 convolution with the final module output. This residual design helps preserve weak signal features and prevent gradient vanishing during dryland seedling detection.

The C2PSA_CGA (see Figure 7b) module replaces the original PSABlock with PSABlock_CGA. It consists of two convolution layers and two PSABlock_CGA modules. The input feature first undergoes dimensionality reduction through a convolution layer, followed by feature extraction via two PSABlock_CGA modules. All intermediate outputs are concatenated along the channel dimension using the Concat operation. Finally, the concatenated feature is processed by another convolution layer to generate the final output. This design enhances the model’s focus on extracting features from occluded weed regions.

## 3. Experimental Results and Analysis

### 3.1. Ablation Study

**Figure 8:**
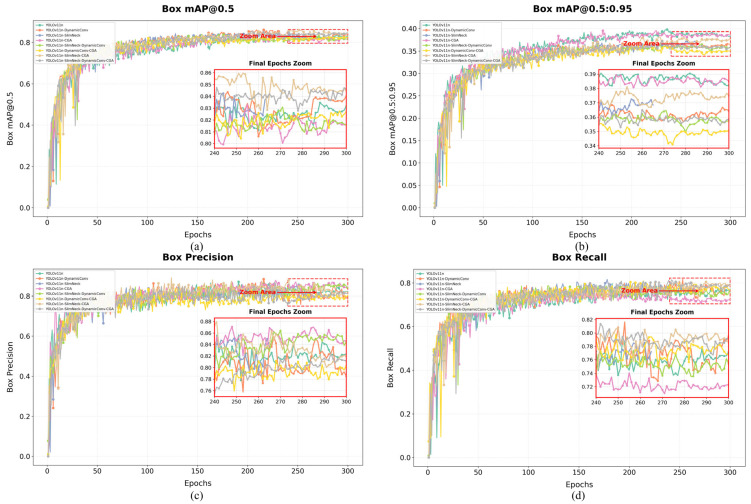
Comparison curves of ablation study results. (**a**) shows the Box mAP@0.5 curves of different models in the ablation study; (**b**) shows the Box mAP@0.5:0.95 curves; (**c**) shows the Box precision curves; and (**d**) shows the Box recall curves for all models evaluated in the ablation experiments.

As shown in Table 1 and Figure 8, the performance differences in various improvement strategies for seedling-stage weed detection under maize–soybean rotation dryland conditions were compared. Overall, the strategies exhibit differences in detection accuracy, model size, and computational complexity. Among them, the proposed DSC-YOLOv11n model (DynamicConv + SlimNeck-2 + CGA) demonstrates the best overall performance.

For the detection task of “broadleaf weeds” and “poaceae weeds” in maize–soybean rotation dryland fields, the comprehensive improvement scheme (Experiment 8) achieves a mAP@0.5(Box) of 0.805, the highest among all ablation experiments. Although the improvement in detection accuracy compared to the combination of only SlimNeck-2 and CGA (Experiment 7, mAP@0.5 0.796) is limited, it significantly outperforms the baseline model (Experiment 1, mAP@0.5 0.766) and most single-strategy improvements. Notably, the detection precision for Poaceae weeds (mAP@0.5 0.739) shows the most significant enhancement, fully demonstrating that the synergistic effects of multiple strategies effectively strengthen the model’s discriminative ability for morphologically similar and difficult-to-recognize weed categories.

From the perspective of model complexity, the comprehensive improvement scheme (Experiment 8) has 2.75 M parameters and a computational cost of 5.6 GFLOPs. Compared with the baseline model (Experiment 1, 2.58 M, 6.3 GFLOPs), the parameters increase slightly, while the computational complexity is significantly reduced. Meanwhile, its scale is comparable to the lightweight model using only SlimNeck-2 (Experiment 3, 2.48 M, 5.8 GFLOPs) and much lower than original or single-attention models with higher computational costs (Experiment 4, 6.3 GFLOPs). This demonstrates that the proposed improvements steadily enhance detection accuracy without significantly increasing—or even reducing—computational overhead, reflecting a good trade-off between efficiency and precision.

Overall, the combination of DynamicConv and SlimNeck-2 (Experiment 5) shows the best efficiency; the SlimNeck-2 and CGA combination (Experiment 7) excels in parameter lightness; and the integration of all three components (Experiment 8) achieves the highest overall detection accuracy. These results confirm the effectiveness of DynamicConv in optimizing computation, SlimNeck-2 in compressing parameters, and CGA in enhancing feature representation.

### 3.2. Ablation Study: Loss Curve Analysis

To evaluate the impact of each improvement module on model performance, the bounding box loss (Box Loss) during the ablation study training process was visualized. Figure 8a shows the bounding box loss curve on the training set (Train Box Loss), while Figure 8b shows the curve on the validation set (Val Box Loss). All models are based on the YOLOv11n baseline, with incremental incorporation of DynamicConv, SlimNeck, CGA, and their combinations, trained for 300 epochs on the maize–soybean rotation dryland seedling-stage weed dataset.

**Figure 9:**
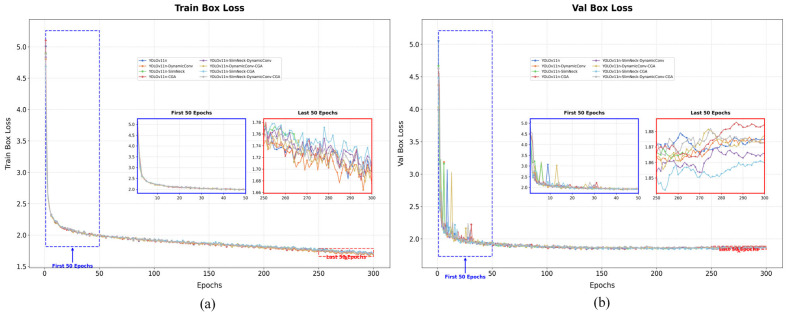
Training and validation loss curves. (**a**) shows the training Box loss curves of different models in the ablation study; (**b**) shows the validation Box loss curves for all models evaluated in the ablation experiments.

From the training loss curves in Figure 9a, it can be observed that the box loss of all models steadily decreases with increasing epochs and begins to converge around 250 epochs. The baseline YOLOv11n model reaches a final loss of approximately 1.5, while models with additional modules show an overall downward shift in the loss curves, indicating enhanced model fitting capability. Among these, the YOLOv11n-DynamicConv-CGA combination exhibits the fastest loss decrease in the early stage (first 50 epochs) and achieves the lowest final loss (close to 1.45), suggesting that the synergy of DynamicConv and CGA effectively accelerates convergence and improves localization accuracy. Models with only SlimNeck show modest improvements over the baseline, but when combined with CGA (YOLOv11n-SlimNeck-CGA), the loss further decreases, confirming the attention mechanism’s effectiveness in enhancing feature extraction.

A zoomed-in view shows that in the first 50 epochs, losses of all models drop rapidly from around 1.78 to approximately 1.66, with models incorporating CGA exhibiting steeper slopes, highlighting CGA’s advantage in early-stage training. In the final 50 epochs, the loss change flattens and inter-model differences narrow, yet improved models maintain lower overall loss levels.

The validation loss curves in Figure 9b reflect the model generalization capability. The overall trend is similar to the training loss, though some models show minor fluctuations in later epochs. From the magnified view of the “Last 50 epochs,” it can be observed that the validation losses of all models are primarily distributed in the range of approximately 1.85–1.88. After 250 epochs, the validation loss of the baseline YOLOv11n is around 1.87, whereas the improved YOLOv11n-SlimNect-DynamicConv-CGA exhibits lower values with a smoother curve, indicating superior generalization performance and a reduced risk of overfitting. Notably, the model with only DynamicConv shows slightly higher validation loss than the SlimNeck version, suggesting that SlimNeck contributes to overfitting suppression. The full model containing DynamicConv, SlimNeck, and CGA achieves the lowest validation loss, further confirming the effectiveness of multi-module integration. Overall, analysis of the training and validation loss curves demonstrates that all improvement modules reduce bounding box loss to varying degrees. CGA significantly contributes to faster convergence, DynamicConv improves localization precision, and SlimNeck enhances generalization ability. The combination of the three modules achieves the best performance during both training and validation, providing a reliable feature extraction foundation for subsequent detection tasks.

### 3.3. Comparative Experiments of Different Models

**Figure 10:**
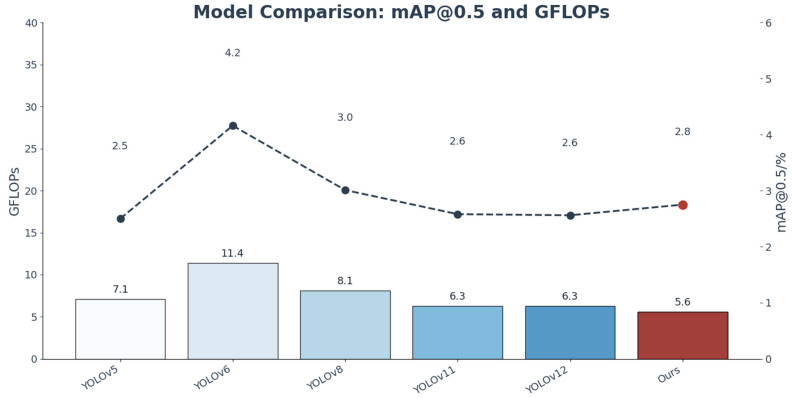
Comparison of YOLO series models on mAP@0.5 and GFLOPs.

As shown in Table 2 and Figure 10, the performance of different lightweight YOLO models was compared for weed detection during the seedling stage in maize–soybean rotation fields. Overall, the models exhibited notable differences in detection accuracy, model size, and computational efficiency, with the proposed DSC-YOLOv11n achieving the most balanced overall performance.

For the task of weed species classification, DSC-YOLOv11n achieved a precision (P) of 0.771, a recall (R) of 0.780, and an average precision at IoU 0.5 (mAP@0.5) of 0.805. The overall mAP@0.5 was the highest among all compared models, significantly outperforming the baseline YOLOv11n (0.766) as well as other lightweight variants (YOLOv5n: 0.757, YOLOv6n: 0.779, YOLOv8n: 0.772, YOLOv12n: 0.729). These results demonstrate that the introduced improvement modules effectively work in synergy, enhancing the model’s comprehensive discrimination and localization capabilities under complex field conditions.

Under the more stringent mAP@0.5:0.95 metric, our model also outperforms the baseline, improving from 0.333 for YOLOv11n to 0.351, indicating an enhanced overall localization capability across multiple IoU thresholds. It should be noted that, compared with mAP@0.5, this metric is more sensitive to bounding box localization accuracy, which explains the relatively modest improvement. Nevertheless, these results demonstrate that our method achieves a steady improvement in localization performance while maintaining its advantages in detection and classification.

Analysis by category reveals that for the well-represented and morphologically distinctive broadleaf weeds all models maintained high precision, with DSC-YOLOv11n achieving an mAP@0.5 of 0.872 nearly identical to the baseline YOLOv11n (0.863). In contrast, for the underrepresented, slender, and crop-like grass weeds—a particularly challenging task that tests model robustness—DSC-YOLOv11n achieved an mAP@0.5 of 0.739, representing a substantial 10.3% improvement over the baseline YOLOv11n (0.670) and significantly outperforming all other compared models. This indicates that the use of the CGA mechanism and other enhancement strategies effectively strengthened the model’s perception and discrimination of subtle features, demonstrating its robustness on the most challenging subtask.

From a model complexity perspective, DSC-YOLOv11n contains 2.75 M parameters and requires only 5.6 GFLOPs of computation. Its parameter count is comparable to mainstream lightweight models (2.50 M–3.01 M), while its computational complexity is the lowest among all compared models, reducing approximately 11.1% relative to the baseline YOLOv11n (6.3 GFLOPs). These findings indicate that the proposed improvements enhance detection accuracy—particularly for grass weeds—without increasing computational load, and even further optimize efficiency, providing an effective foundational model for variable-rate weed management during the seedling stage.

The primary objective of this study is to support weed species recognition under corn–soybean rotation conditions. In practical applications, accurately distinguishing between different weed types (e.g., broadleaf versus grass species) is more critical for subsequent precision herbicide application than achieving pixel-level localization accuracy. Therefore, mAP@0.5 remains an important reference metric for evaluating the model’s classification and detection capabilities. Although mAP@0.5:0.95 provides a more stringent assessment of localization performance, our method also achieves a stable improvement under this metric, validating its overall effectiveness.

In summary, DSC-YOLOv11n achieves higher accuracy while retaining lightweight characteristics, with particularly notable improvements in detecting challenging categories (Poaceae weeds) and reducing computational overhead. The experimental results validate the effectiveness of the synergistic design of DynamicConv, SlimNeck-2, and CGA modules, making the model well-suited for resource-constrained lightweight UAV platforms and enabling real-time, precise weed identification in maize–soybean rotation fields during the seedling stage.

**Figure 11:**
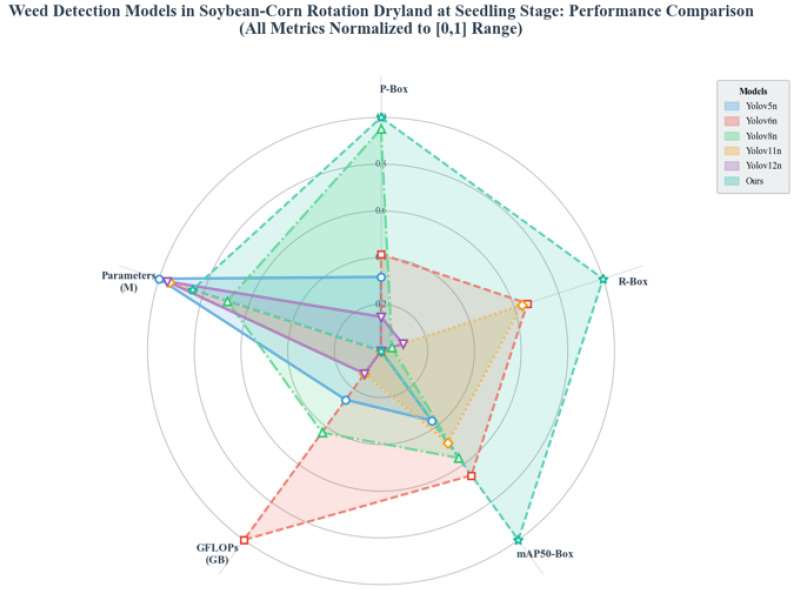
Comparison radar chart of models.

To further verify the comprehensive performance of the improved DSC-YOLOv11n (Ours) model for seedling weed detection in corn–soybean rotation dry fields, it was compared with mainstream lightweight object detection models, including YOLOv5n, YOLOv6n, YOLOv8n, YOLOv11n, and YOLOv12n, from multiple dimensions. Figure 11 intuitively shows the relative performance of each model in core detection metrics, including P-Box, R-Box, mAP50-Box, and mAP50-95-Box, as well as parameters (M) and GFLOPs. All indicators were normalized to the range of (0, 1) to facilitate direct cross-model comparison.

According to the radar chart and quantitative results, our model achieves overall superiority in core detection accuracy. The overall mAP50-95 reaches 0.805, which is significantly higher than baseline models such as YOLOv11n (0.766) and YOLOv8n (0.772). Compared with YOLOv11n, our model improves by approximately 5.1%, and by about 10.4% compared with YOLOv12n (0.729), which has a similar number of parameters. For fine-grained classification, the mAP50-95 values of broadleaf weeds and Poaceae weeds are 0.872 and 0.739, representing improvements of 1.0% and 10.3% over YOLOv11n, respectively. Our model notably enhances the detection performance of Poaceae weeds, the minority class, and effectively alleviates the accuracy degradation caused by class imbalance in the dataset.

In terms of model efficiency, the proposed model maintains excellent lightweight properties. Its parameters are only 5.6 M, which is lower than YOLOv8n (8.1 M) and YOLOv6n (11.4 M), and about 11.1% less than the baseline YOLOv11n (6.3 M). The GFLOPs value is 2.75 GB, which is comparable to YOLOv11n (2.58 GB) and YOLOv5n (2.50 GB), and much lower than YOLOv6n (4.16 GB). This demonstrates that the introduced improvements do not introduce obvious computational overhead and maintain efficient inference while improving detection accuracy.

Based on the radar chart and quantitative indicators, DSC-YOLOv11n achieves an optimal trade-off between accuracy and efficiency for seedling weed detection in dry fields. Our model occupies the outermost contour in all four precision dimensions: P-Box, R-Box, mAP50-Box, and mAP50-95-Box, while maintaining low parameters and GFLOPs. The collaborative integration of DynamicConv, SlimNeck-2 feature fusion, and CGA context attention effectively strengthens feature representation for small targets under complex backgrounds and preserves lightweight advantages. The proposed model is particularly suitable for deployment on resource-constrained agricultural mobile devices, providing reliable support for real-time weed recognition and precision herbicide application in future applications.

### 3.4. Detection Performance of the DSC-YOLOv11n Model

**Figure 12:**
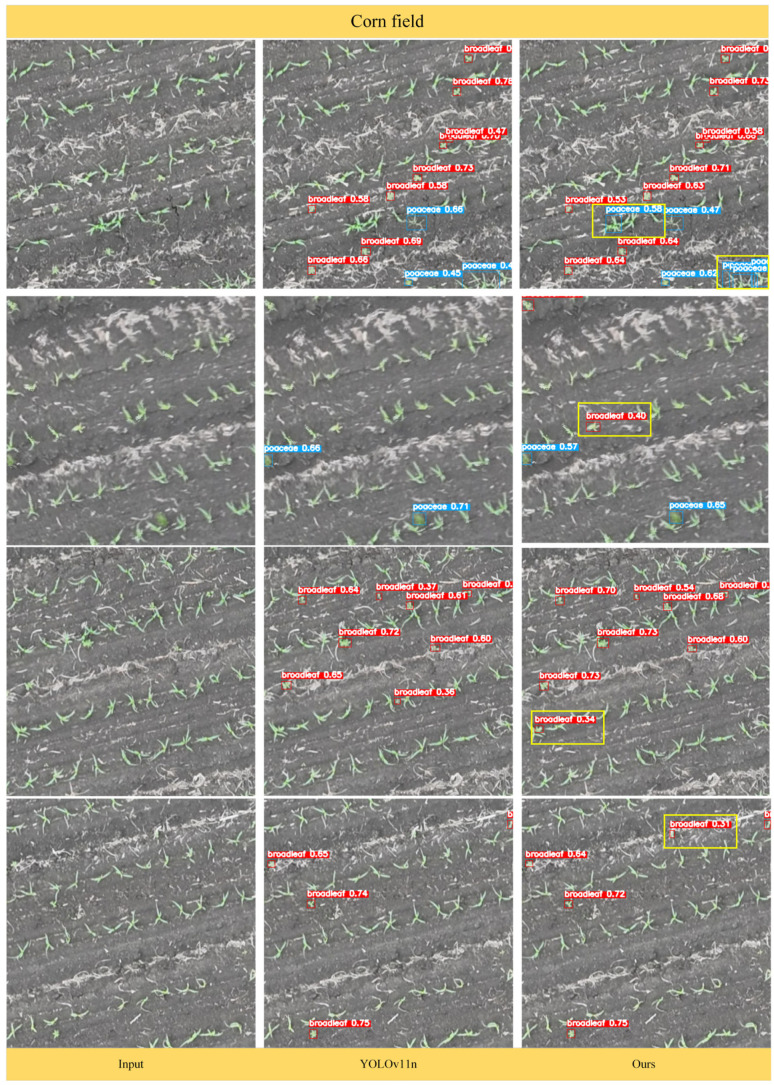
Comparison of the detection performance of the improved DSC-YOLOv11n model in cornfields. Note: the yellow boxes highlight the differences between the detection results of DSC-YOLOv11n and YOLOv11n in maize fields.

**Figure 13:**
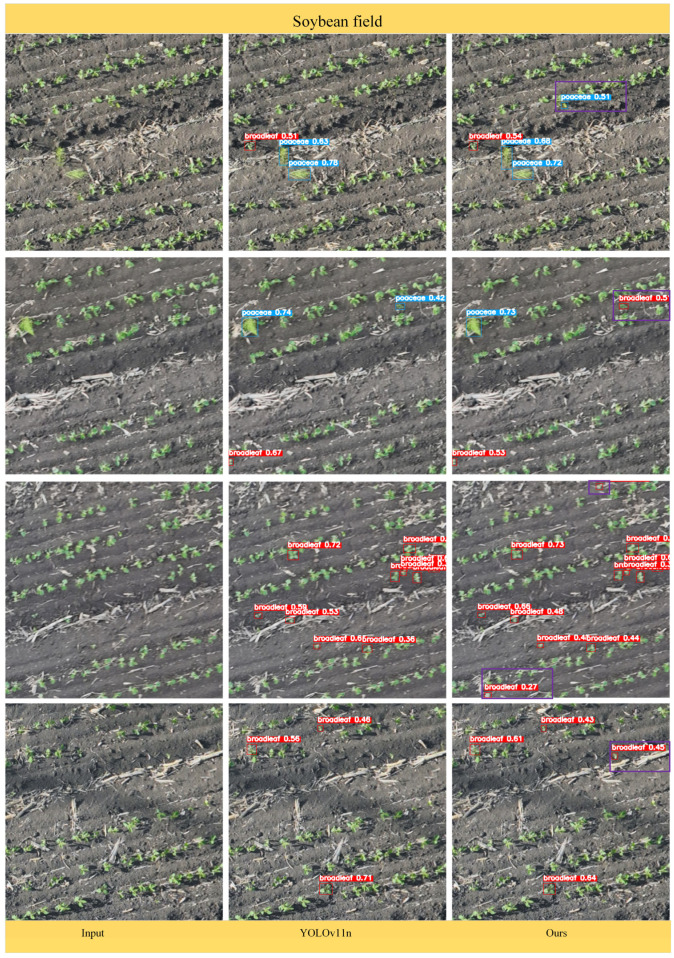
Comparison of the detection performance of the improved DSC-YOLOv11n model in soybean fields. Note: the purple boxes highlight the differences between the detection results of DSC-YOLOv11n and YOLOv11n in soybean fields.

To more intuitively evaluate the performance of the DSC-YOLOv11n algorithm, this study visualized its results on maize–soybean rotation field seedling weed detection. As shown in Figure 12 and Figure 13, the model accurately detects and localizes weeds under complex field conditions, including small-sized weeds, mutually occluded weeds, densely clustered weed patches, and objects with morphologies similar to maize and soybean seedlings. The model effectively differentiates Poaceae (grasses) and broadleaf weeds, with bounding boxes precisely aligning to targets, avoiding overfitting or missed detections. Analysis of the visualizations shows that DSC-YOLOv11n demonstrates high robustness in handling complex field scenarios, such as crop residue, soil texture, and target overlap.

Specifically, in maize fields, the original YOLOv11n exhibited lower detection confidence for certain weed targets and occasional missed detections. In contrast, DSC-YOLOv11n reliably detected all weeds, with higher overall confidence scores and more accurate classification of both Poaceae and broadleaf weeds, effectively preventing misidentification of crop seedlings. In soybean fields, YOLOv11n struggled with densely distributed small weeds, often missing targets or showing insufficient confidence. DSC-YOLOv11n, through optimized feature extraction and multi-scale detection heads, significantly improved detection of small weeds, dense clusters, and occluded weeds, while maintaining high detection confidence (e.g., 0.51, 0.61), achieving precise distinction between weeds and crop seedlings even in complex backgrounds.

Compared with the original YOLOv11n, DSC-YOLOv11n not only increases detection confidence but also effectively suppresses misclassification of crop seedlings, demonstrating superior environmental adaptability in both maize and soybean rotation scenarios. These visualizations confirm the reliability and practicality of the improved YOLOv11n for real-world agricultural applications, providing effective technical support for precision weed management.

To quantitatively and qualitatively verify DSC-YOLOv11n’s actual performance, experiments were conducted on a self-built maize–soybean rotation weed dataset. Visualizations from maize and soybean fields indicate that DSC-YOLOv11n significantly outperforms the baseline YOLOv11n in terms of detection completeness, bounding box localization accuracy, and adaptability to complex backgrounds. In maize fields, where weed and seedling morphologies are highly similar, DSC-YOLOv11n consistently detects broadleaf and Poaceae weeds occluded by soil or crop residue, with bounding boxes tightly fitting the target contours and a noticeably lower misdetection rate for low-confidence targets. In soybean fields, where seedlings are densely planted, and weeds spatially overlap with crops, the model reduces target confusion and accurately detects weeds across scales, showing particularly strong discrimination in densely populated areas. Quantitative comparison results demonstrate that DSC-YOLOv11n achieves Precision 77.1%, Recall 78.0%, mAP@0.5 80.5%, and mAP@0.5–0.95 35.1% for all classes, outperforming mainstream models including YOLOv5, YOLOv6, YOLOv8, YOLOv11, and YOLOv12. Specifically, mAP@0.5 for broadleaf weeds and Poaceae weeds reaches 87.2% and 73.9%, respectively, while maintaining low computational cost (5.6 GFLOPs) and moderate parameter count (2.75 M), achieving a balance between accuracy and efficiency. Overall, by integrating DynamicConv for adaptive convolution, SlimNeck for lightweight feature fusion, and CGA for context-guided attention, DSC-YOLOv11n significantly enhances detection accuracy and robustness in maize–soybean rotation fields. It exhibits superior target discrimination under complex lighting, occlusion, and background interference, providing reliable technical support for variable-rate herbicide application and precision field management.

### 3.5. Feature Map Visualization Analysis

To validate the feature extraction and target localization capabilities of DSC-YOLOv11n in maize–soybean rotation field seedling weed detection, this study employed Gradient-weighted Class Activation Mapping (Grad-CAM) to visualize the backbone network’s output feature maps [25]. Representative images from maize and soybean seedling fields were selected as inputs to compare the feature response differences between the original YOLOv11n and the improved model.

**Figure 14:**
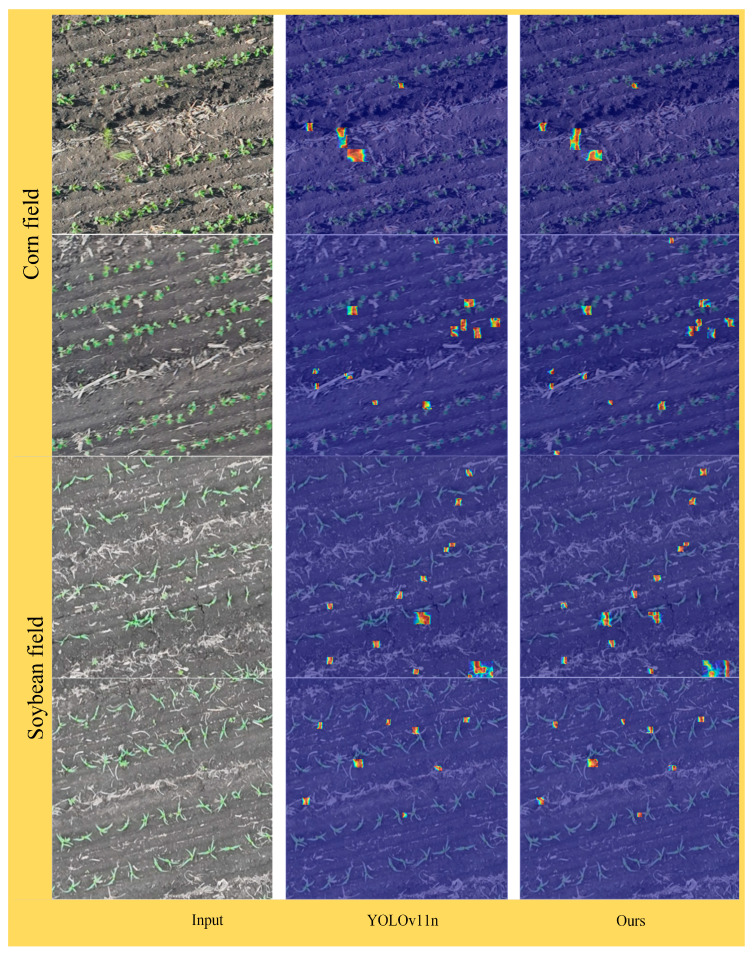
Grad-CAM feature map analysis.

As shown in Figure 14, in maize field scenarios, the original YOLOv11n exhibited multiple scattered high-activation regions, some of which were misaligned with actual weed targets, indicating noticeable background interference. In contrast, the improved model’s feature responses were precisely concentrated on weed locations, effectively suppressing responses to crop seedlings and soil background. Similarly, in soybean field scenarios, the original YOLOv11n showed weak and blurry feature responses for certain weed targets. DSC-YOLOv11n, however, produced more focused and well-defined feature responses, accurately localizing the target regions. Both scenarios included complex background noise, such as crop residues and soil textures. The original YOLOv11n feature maps contained numerous scattered activation points in these background areas, whereas DSC-YOLOv11n, with its attention mechanisms, effectively suppressed background interference. The feature responses were predominantly concentrated on weed targets, with significantly reduced activation in background regions, enhancing the model’s robustness in complex field environments.

Moreover, the improved model preserved both local texture details (e.g., leaf edges) and overall contours of the weeds. This indicates that its backbone network effectively fused multi-scale features: low-scale features captured fine weed textures, mid-scale features highlighted overall weed contours, and high-scale features focused on semantic information. Such multi-scale feature integration provides more reliable support for accurate classification and localization by subsequent detection heads.

In summary, the feature map visualization analysis confirms that the improved DSC-YOLOv11n model possesses precise target localization, stronger resistance to background interference, and enhanced multi-scale feature fusion for maize–soybean rotation seedling weed detection. These results provide visual evidence of the model’s effectiveness in practical field applications and serve as an important reference for further optimization of the network architecture.

## 4. Discussion

### 4.1. Analysis of DSC-YOLOv11n

The seedling stage represents a critical period for implementing precision weeding and variable-rate herbicide application. However, in maize–soybean rotation dryland fields, weed targets are generally small, and Poaceae weeds often exhibit morphological similarities to maize seedlings. Coupled with complex soil backgrounds and varying illumination, accurate recognition and localization pose significant challenges [26,27]. To address these issues, this study developed the DSC-YOLOv11n model and validated it on a self-constructed field dataset.

Overall, under the experimental conditions and data distribution used, DSC-YOLOv11n achieved a mAP@0.5 of 0.805 on the test set, representing a 3.9% improvement over the baseline model. Class-specific results indicated that detection performance for broadleaf weeds generally exceeded that for Poaceae weeds, the latter being more prone to false positives and missed detections due to their slender leaves and high structural similarity. Notably, the improved model demonstrated a more significant mAP@0.5 gain for Poaceae weeds, suggesting that the architectural modifications enhanced fine-grained feature discrimination [28].

Ablation experiments further revealed the contribution of each module. Firstly, the introduction of DynamicConv improved the model’s adaptability to morphologically variable weeds without significantly increasing computational complexity. Dynamic convolution adaptively adjusts convolutional kernel weights based on input features, enabling the network to generate more targeted feature responses under varying scales and textures, thereby enhancing recognition of small targets in complex backgrounds. Experiment results showed a stable upward trend in overall mAP after incorporating DynamicConv [29].

Secondly, the SlimNeck-2 structure positively influenced model lightweighting during feature fusion. Compared with the baseline, the improved model maintained a GFLOPs of approximately 5.6 and a parameter count of 2.75 M. Despite the reduced or comparable computational load, detection accuracy did not degrade, indicating that the optimized feature fusion path preserves multi-scale semantic information while compressing computation. From a deployment perspective, this structure is potentially advantageous for UAVs or embedded devices.

Additionally, the CGA module improved training stability. Training and validation loss curves demonstrated faster early-stage convergence and reduced fluctuations in validation loss with CGA [30], indicating enhanced responsiveness to critical regions and reduced interference from background noise. However, its contribution to final accuracy metrics was relatively modest, manifesting more in stabilized training and improved robustness.

Through the combined effect of these modules, DSC-YOLOv11n achieved gradual performance improvements while maintaining a lightweight architecture. Compared with some larger models, this study’s model strikes a relative balance between computational complexity and detection accuracy. It should be noted, however, that these performance gains were obtained under specific data scale and collection conditions, and performance in more complex environments requires further validation.

### 4.2. Trade-Off Between Accuracy and Efficiency and Its Practical Significance

In agricultural applications, balancing model accuracy and computational efficiency is of practical significance [31]. UAVs or field operation platforms typically impose limitations on inference latency and power consumption, making model size and computational complexity critical. The current study demonstrates that lightweight structural optimization can achieve stable accuracy improvements while keeping parameters and GFLOPs low, indicating feasibility for real-world field tasks.

In current practical applications of weed management during the seedling stage in agricultural fields, unmanned aerial vehicles (UAVs) are mostly used to first collect field images, which are then processed and analyzed offline after being transferred to the laboratory, rather than relying on real-time inference on the airborne platform. Therefore, the core objective of this study is to improve the accuracy and reliability of weed detection as much as possible, so as to provide a solid basis for decision-making in precise weed control [32].

It should be noted that GFLOPs reflect theoretical computation and do not directly equate to actual inference time. Performance may vary across different hardware platforms (e.g., GPUs, embedded chips, UAV processors). Therefore, future research should include real deployment evaluations to comprehensively assess inference speed and energy consumption [33,34,35].

### 4.3. Limitations and Future Work

Despite the improvements achieved, several limitations exist. First, data collection was concentrated at a single farm over a limited time span, with relatively homogeneous environmental conditions, leaving cross-regional generalization unverified. Second, only two weed categories were considered, limiting applicability under more diverse weed communities. Third, the evaluation focused primarily on detection metrics, without in-depth analysis of spatial continuity or row-level localization errors. Future work may explore: (1) Constructing multi-region, multi-year datasets to systematically evaluate generalization. (2) Integrating instance segmentation or fine-grained segmentation methods to better handle dense small targets. (3) Incorporating spatial localization errors and row-level reliability analysis to enhance interpretability for precision herbicide application. (4) Conducting real-world UAV or edge-device deployment tests to validate engineering feasibility.

## 5. Conclusions

In this study, a novel lightweight weed detection model, DSC-YOLOv11n, was developed specifically for seedling-stage weed monitoring in maize–soybean rotation dryland fields, aiming to provide accurate and efficient detection of both broadleaf and Poaceae weeds under complex field conditions. By integrating DynamicConv for adaptive feature extraction, SlimNeck-2 for lightweight multi-scale feature fusion, and the CGA contextual attention mechanism for enhancing critical region responses, the proposed model effectively addresses challenges posed by small target size, morphological similarity between crops and weeds, dense planting, occlusion, and heterogeneous soil backgrounds. Experimental results on a self-constructed maize–soybean rotation seedling dataset demonstrated that DSC-YOLOv11n achieves an overall mAP@0.5 of 80.5%, with class-specific mAP@0.5 values of 87.2% for broadleaf weeds and 73.9% for Poaceae weeds, representing substantial improvements over baseline YOLOv11n and other mainstream lightweight models (YOLOv5n, YOLOv6n, YOLOv8n, YOLOv12n) in both detection accuracy and fine-grained target discrimination. Notably, the introduction of DynamicConv allowed the network to generate adaptive feature responses to varying weed scales and morphologies, SlimNeck-2 optimized multi-scale feature fusion while maintaining a low computational cost (5.6 GFLOPs) and compact parameter size (2.75 M), and CGA effectively enhanced feature robustness and suppressed background interference, especially in occluded or overlapping regions. Visualizations using Grad-CAM further confirmed that the model concentrates feature responses on weeds while reducing background noise and maintaining fine-grained texture and contour information. The combination of these modules thus enables DSC-YOLOv11n to maintain a superior balance between precision, computational efficiency, and robustness, making it highly suitable for deployment on UAVs and other resource-constrained edge devices for real-time weed detection. Overall, this research provides a practical and scalable approach for precision weed management, offering reliable technical support for variable-rate herbicide application, reducing chemical usage and production costs, mitigating environmental impact, and promoting intelligent, sustainable agricultural practices.

## Figures and Tables

**Table 1:** Ablation study results for different improved modules.

Test Umber	DynamicConv	SlimNeck-2	CGA	Task	P(%)-Box	R(%)-Box	mAP@0.5(%)-Box	mAP@0.5-0.95(%)-Box	GFLOPs (GB)	Parameters (M)
1				all classes	0.73	0.765	0.766	0.333	6.3	2.58
				broadleaf	0.822	0.834	0.863	0.357		
2	**√**			poaceae	0.638	0.697	0.67	0.31	5.9	3.23
				all classes	0.72	0.761	0.785	0.343		
				broadleaf	0.801	0.835	0.867	0.361		
3		**√**		poaceae	0.64	0.687	0.703	0.325	5.8	2.48
				all classes	0.802	0.714	0.792	0.342		
				broadleaf	0.858	0.791	0.865	0.357		
4			**√**	poaceae	0.746	0.638	0.718	0.327	6.3	2.56
				all classes	0.793	0.711	0.776	0.364		
				broadleaf	0.848	0.762	0.825	0.363		
5	**√**	**√**		poaceae	0.738	0.66	0.727	0.365	5.6	2.78
				all classes	0.76	0.754	0.789	0.349		
				broadleaf	0.847	0.824	0.877	0.36		
6	**√**		**√**	poaceae	0.673	0.685	0.7	0.338		
				all classes	0.755	0.771	0.78	0.341	5.9	3.2
				broadleaf	0.821	0.841	0.871	0.361		
				poaceae	0.69	0.701	0.689	0.321		
7		**√**	**√**	all classes	0.792	0.735	0.796	0.342	5.8	2.46
				broadleaf	0.861	0.795	0.88	0.36		
				poaceae	0.723	0.674	0.712	0.325		
8	**√**	**√**	**√**	all classes	0.771	0.78	0.805	0.351	5.6	2.75
				broadleaf	0.851	0.811	0.872	0.362		
				poaceae	0.691	0.748	0.739	0.34		

‘**√**’ indicates the method is applied.

**Table 2:** Algorithm comparison of test results.

Model	Task	P(%)-Box	R(%)-Box	mAP@0.5(%)-Box	GFLOPs (GB)	Parameters (M)
YOLOv5	all classes	0.743	0.739	0.757	7.1	2.50
	broadleaf	0.824	0.773	0.812		
	poaceae	0.662	0.706	0.703		
YOLOv6	all classes	0.747	0.766	0.779	11.4	4.16
	broadleaf	0.824	0.811	0.86		
	poaceae	0.671	0.721	0.699		
YOLOv8	all classes	0.769	0.741	0.772	8.1	3.01
	broadleaf	0.836	0.774	0.824		
	poaceae	0.703	0.708	0.72		
YOLOv11	all classes	0.73	0.765	0.766	6.3	2.58
	broadleaf	0.822	0.834	0.863		
	poaceae	0.638	0.697	0.67		
YOLOv12	all classes	0.736	0.743	0.729	6.3	2.56
	broadleaf	0.821	0.765	0.819		
	poaceae	0.65	0.72	0.64		
Ours	all classes	0.771	0.78	0.805	5.6	2.75
	broadleaf	0.851	0.811	0.872		
	poaceae	0.691	0.748	0.739		

## Data Availability

The original contributions presented in this study are included in the article. Further inquiries can be directed to the corresponding author.
